# Pelvic Venous Congestion Secondary to a Circumaortic Renal Collar in an Adolescent Female: Report of a Case

**DOI:** 10.1055/s-0041-1730998

**Published:** 2021-08-10

**Authors:** Verónica Alonso-Arroyo, Jose Javier Velasco, Sonia Pérez-Bertólez, Maria Elena Molina, Jose Manuel Marugan-de-Miguelsanz, Alberto Sanchez-Abuin, Oscar Dario Gomez Beltran

**Affiliations:** 1 Department of Pediatric Surgery, Hospital Clinico Universitario de Valladolid, Valladolid, Castilla y León, Spain; 2 Department of Interventional Radiology, Hospital Clinico Universitario de Valladolid, Valladolid, Castilla y León, Spain; 3 Department of Pediatric Urology, Hospital Sant Joan de Deu, Barcelona, Catalunya, Spain; 4 Department of Pediatric Gastroenterology, Hospital Clinico Universitario de Valladolid, Valladolid, Castilla y León, Spain

**Keywords:** renal vein, pelvic pain, embolization, phlebographie

## Abstract

We report a 13-year-old girl who presented with a recurrent abdominal pain that started after her menarche. The abdominal palpation revealed tenderness over the left ovarian point. The laboratory study, ultrasonography, and abdominal X-ray were normal. The computed tomography and magnetic resonance imaging showed a double left renal vein with a retroaortic component, an increased left parauterine circulation, and ipsilateral ovarian vein engorgement. A diagnostic and therapeutic phlebography allowed a selective catheterization of a group of pelvic varicose veins draining to the left ovarian and to the internal iliac veins. There were no complications during the procedure and the symptoms disappeared 2 days later. Circumaortic left renal vein may cause hematuria, proteinuria, pelvic congestion syndrome, and massive hemorrhage during surgery. A conservative treatment is recommended for patients without gynecourological/renal symptoms or with mild hematuria. The endovascular treatment by gonadal venous embolization is safe and effective.

## Introduction

Normal anatomical relationships between renal vessels, abdominal aorta and inferior cava vein (ICV), as well as variants of the renal arteries and veins were illustrated for the first time in 1564 in the Eustachi's treatise titled *Opuscula anatomica* . In this article, we will focus on the left circumaortic renal vein or venous collar.

Left renal vein (LRV) anatomy has a complex embryological development, and a failure in its formation may originate venous anomalies including circumaortic or retroaortic variants. During the fetal stage, the initial venous channels undergo several phases of remodeling; afterward, a series of anastomoses between the ventral subcardinal and dorsal supracardinal veins causes a renal collar around the aorta. Usually, the dorsal branch degenerates, resulting in a single LRV. The existence of a circumaortic renal vein or venous collar is explained by the persistence of both branches. 1 As far as we know, there are just five pediatric cases (< 18 years old) of this last variant reported in the literature. 2 3 4 5 6

This rare anomaly can cause hematuria and proteinuria (secondary to the subsequent nutcracker phenomenon), pelviureteric junction obstruction, as well as pelvic congestion syndrome in women or varicocele in men. 7

## Case Report

**Fig. 1:**
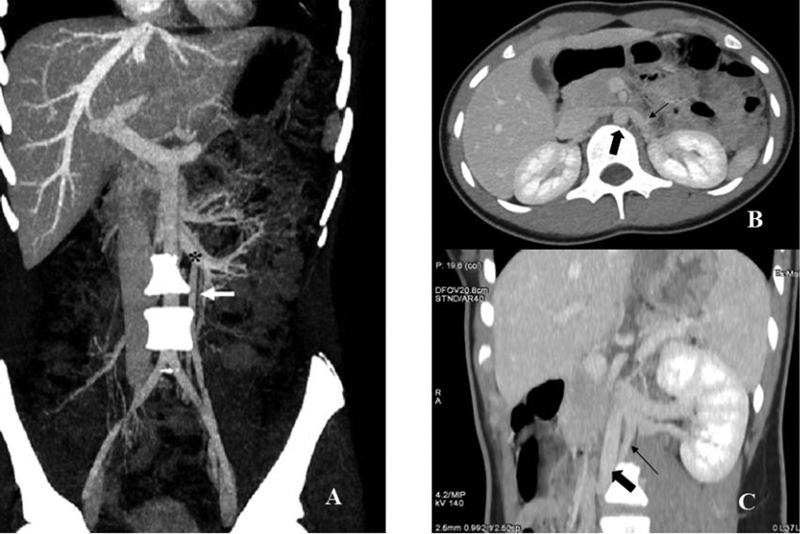
Abdominal pelvic computed tomography scan with contrast. ( **A** ) Coronal plane showing a double left renal vein (LRV) with a retroaortic component (black star) caudal to the main renal vein and a dilated left ovarian vein (white arrow). ( **B** ) Transversal plane showing the anterior branch of the LRV (thin arrow) and the aorta artery (thick arrow). ( **C** ) Coronal plane showing the posterior branch of the LRV (thin arrow) and the aorta artery (thick arrow).

**Fig. 2:**
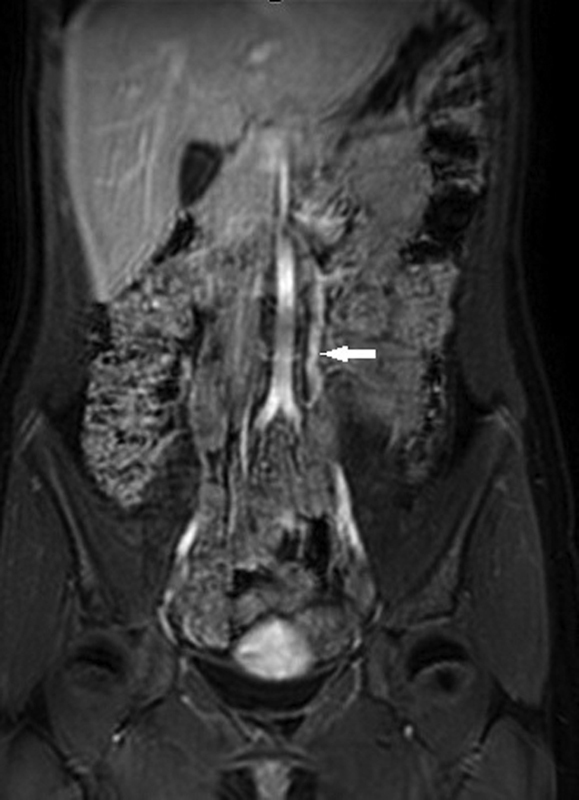
Magnetic resonance imaging showing a dilated left ovarian vein (white arrow) with a caliber of 8 mm and a grade II reflux, draining into the retroaortic component of the left renal vein.

A 13-year-old girl presented with recurrent abdominal pain for the last 3 months that appeared after menarche. The pain negatively affected her quality of life including a decrease in her school attendance, forcing her to go to the emergency room on several occasions. The physical examination revealed tenderness over the left ovarian point with no signs of peritoneal irritation. The patient was evaluated by a multidisciplinary team made up of pediatric specialists in gastroenterology, surgery, nephrology, and radiology. Laboratory tests, including complete blood count, serum biochemistry, urinary sediment, biochemical test for renal function, celiac disease serology, fecal parasites, and calprotectin, were normal. The abdominal X-ray was also normal. The abdominal ultrasound did not reveal any abnormalities including both kidneys; however, intestinal gas prevented a complete visualization of the uterus and ovaries. Finally, the computed tomography with intravenous contrast ( Fig. 1 ) and magnetic resonance imaging ( Fig. 2 ) showed a double LRV with a retroaortic component, prominent left parauterine circulation and engorgement of the ipsilateral ovarian vein with a caliber of 8 mm and a grade II reflux. These findings were consistent with pelvic venous congestion (PVC) secondary to a circumaortic LRV with a posterior nutcracker phenomenon.

**Fig. 3:**
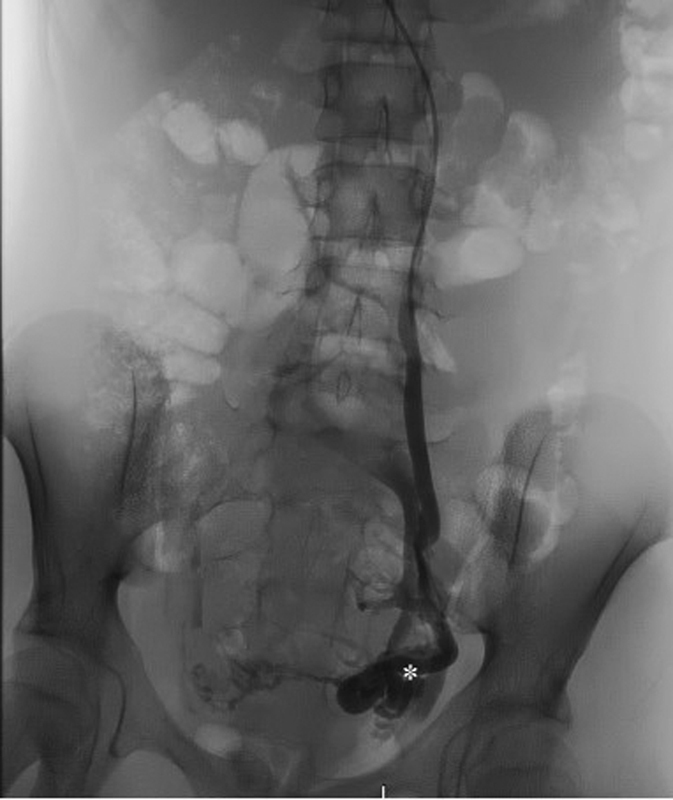
Venography image showing a group of pelvic varicose veins (white star) with a maximum caliber of 8 to 9 mm, draining into the left ovarian and internal iliac veins.

**Fig. 4:**
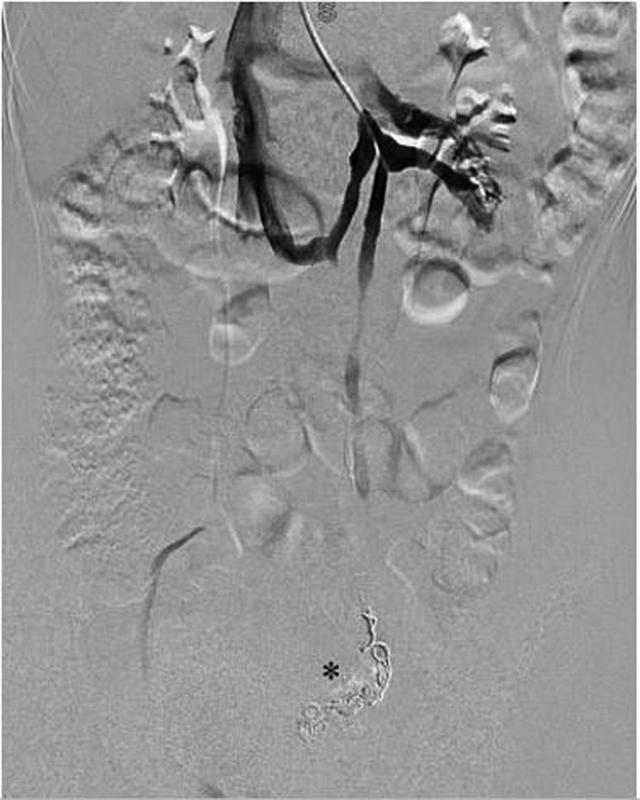
Venography image showing the circumaortic left renal vein and the Hydrocoils (black star) used for the parauterine and left ovarian veins embolization.

A diagnostic and therapeutic phlebography was programmed. This procedure was performed through the right basilic vein and confirmed the existence of a left circumaortic renal vein and a group of dilated pelvic varicose veins draining into the left ovarian and internal iliac veins ( Fig. 3 ). An embolization at a distal level of these vessels was accomplished using 8 to 10 mm Hydrocoils under a 0.035” guide ( Fig. 4 ). The complete occlusion of the pelvic varices and the treated venous segment was confirmed with a final angiographic control. There were no perioperative complications. The patient reported moderate pelvic pain, which responded promptly to parenteral analgesia and ceased during the postoperative day 3, remaining asymptomatic after 18 months of follow-up. Urinary tests were requested at 1, 3, 6, and 12 months in which result was normal.

## Discussion

Renal vessels demonstrate a huge anatomical variability as regard to their number, level of origin, diameter, and topographical relationships. Some studies have confirmed that the frequency of renal vein variations is significantly higher on the right side. 8

*Congenital anomalies* of the LRV are classified into four types. In type I, the ventral preaortic limb of the LRV is obliterated, the dorsal retroaortic limb persists and joins the IVC in the orthotopic position. Type II anomaly results from obliteration of the ventral limb of the LRV, and the dorsal limb joins the gonadal and ascending lumbar veins at the level of L4–5, before joining the IVC. Type III anomaly is the circumaortic LRV as in our patient. In type IV, the ventral preaortic limb of the LRV is obliterated, and the remaining dorsal limb becomes the retroaortic LRV and joins the left *common iliac vein* .

The incidence of venous collar is low, although it differs from 0.3 to 8.7% depending on the author. 9 Despite this, if all small retroaortic veins that empty into the ICV are considered, the incidence of a circumaortic LRV could be as high as 16%. 7

It occurs with a similar frequency in both sexes and may be accompanied by atypical topographical relationships between renal vessels, as well as by the possibility of cooccurrence of arterial variations. 9

Knowledge of these anatomical variants may be crucial during retroperitoneal surgery and renal transplants to avoid complications such as massive hemorrhage. 10 On further consideration, the clinical importance of a circumaortic LRV is the presence of a posterior or anterior nutcracker phenomenon. The correlation between imaging evidence of this vascular anomaly and clinical manifestations remains challenging. Intervention should be considered only when symptoms are severe or persistent, including pain, hematuria, renal insufficiency, hypertension, and failure to respond to conservative treatment. 11 In our patient, the recurrent pain made us raise the need for a treatment.

Surgical procedures employed to rectify the problem include transposition of the LRV, renal autotransplantation, gonadocaval bypass, and extravascular stents. Alternatively, minimally invasive options are intravascular stents placement and intrapelvic chemical cauterization or coil embolization. 12 The use of endovascular techniques has been extrapolated from their use in the anterior nutcracker syndrome. However, there are very few reported cases on the application of stents in the posterior nutcracker syndrome. 13 We agree with other groups in the fact that this location unnecessarily predisposes young patients to an aorto-LRV fistula or potential stent erosion into the spine. 14

Focusing now on the PVC, it occurs when varicose veins develop around the many pelvic organs accompanied by certain angiographic criteria, specifically; incompetent pelvic veins with 5 to 10 mm diameters, reflux, flow stasis in the ovarian venous plexus, and visualization of these vessels in the mid pelvic line (vulvovaginal) and upper thighs. 15

There are two main factors that appear to play an important role in the origin of this congestion. The first is venous incompetence caused by congenital dysfunctional or absence of valves, and the second is the increase in pelvic venous capacity with subsequent hypertension and retrograde flow. 16 This last one is secondary to mechanical compression of the renal vein (nutcracker syndrome) or the left iliac vein (May–Thurner syndrome). 17

The cause of pain remains unclear, but the most likely possibility is that increased dilatation, concomitant with stasis, leads to the release of local pain- producing mediators. In our patient, the retroaortic component of the circumaortic LRV caused an obstruction in the drainage of the ipsilateral ovarian vein and consequently a PVC and recurrent pelvic pain. In addition to these anatomical factors, hormonal changes may also be related, giving an explanation to our patient's onset of discomfort after menarche.

Conventional treatments that have been suggested for PVC include total abdominal hysterectomy, ligature or occlusion of pelvic varicose veins, and hormone therapy. Medroxyprogesterone acetate temporarily decreases pain scores blocking the direct vasodilator effect of estrogen and reducing venous distention; unfortunately, it has also been associated with side effects such as weight gain and acne. Pelvic venous ligature is rarely used nowadays and total abdominal hysterectomy is unacceptable in young women. Finally, although the studies available are of low quality, the endovascular approach, with embolization of pelvic varicose veins and points of reflux, seems to be the best therapeutic method for this disorder. This occlusion can be done with steel or platinum coils alone or in combination with sclerosants. 15 17 18 19 Reported therapeutic success rates of embolization to treat PVC range from 70 to 85%, with no negative impacts on the menstrual cycle, fertility, or ovarian hormone levels. Complications such as coils migration, incapacitating pain, venous rupture and postural hypotension are estimated at 3.4 to 9%. 15

Controversy remains concerning treatment of upstream obstruction when PVC is secondary to nutcracker or May–Thurner syndromes. In our case, we decided to treat the consequence of the circumaortic LRV, which was the PVC, instead of treating the renal vascular anomaly, since her renal function was not affected and a normal anterior left renal branch was present to drain this kidney. To treat her symptoms, the embolization of the varicose veins was chosen for being safe, less invasive, easier, and widely used by our interventional radiology service. Pain recurrence has been reported in 10 to 30% of patients after initial embolization 20 21 in that case, the endovascular procedure can be repeated. Although this method was successful, we must continue to do nephrourological controls to detect potential renal problems that may need additional techniques. Imaging tests would be recommended if laboratory tests are altered or the pain recurs.

## Conclusions

Circumaortic LRV is a rare vascular anomaly that may cause hematuria, proteinuria, pelvic congestion syndrome, and massive hemorrhage during surgery. A conservative treatment is recommended for patients without gynecourological/renal symptoms or mild hematuria.

The endovascular treatment of PVC by gonadal venous embolization is safer than other techniques, effective, and leads to a significant improvement in pain. However, when this congestion is secondary to a left renal collar, the upstream obstruction must be followed up as additional interventions might be needed.

## Notes

New Insights and the Importance for the Pediatric Surgeon Circumaortic left renal vein or left venous collar is a rare vascular anomaly and an infrequent cause of pelvic venous congestion. This case report highlights the success of minimally invasive management.
